# Towards a more accurate global picture of perimenopause

**DOI:** 10.2471/BLT.24.292659

**Published:** 2024-11-06

**Authors:** Yvette Pyne, Jo Burgin, Martha Hickey

**Affiliations:** a Centre for Academic Primary Care, University of Bristol, 39 Whatley Rd, Clifton, Bristol, BS8 2PS, England.; b Department of Obstetrics, Gynaecology and Newborn Health, University of Melbourne and the Royal Women’s Hospital, Melbourne, Australia.

Perimenopause is a life stage that usually precedes spontaneous menopause – that is, when menstruation ceases. Understanding how people perceive and manage perimenopause is vital both to help women (and other people born with ovaries) prepare, and to support health workers to meet their needs. However, diagnosing perimenopause is challenging.

The Stages of Reproductive Aging Workshop; (STRAW +10 classification^1^; Fig. 1) defines reproductive stages, including perimenopause, focusing on changes in menstrual cycle and follicle stimulating hormone levels. While consistent high levels of this hormone can definitively diagnose menopause, they can be in a normal range in perimenopause. Additionally, exclusively using these characteristics to define the perimenopausal stage is problematic for women taking hormonal contraceptives or those who have had surgery that affects their menstrual cycle, and for women who lack access to blood tests.

**Fig. 1:**
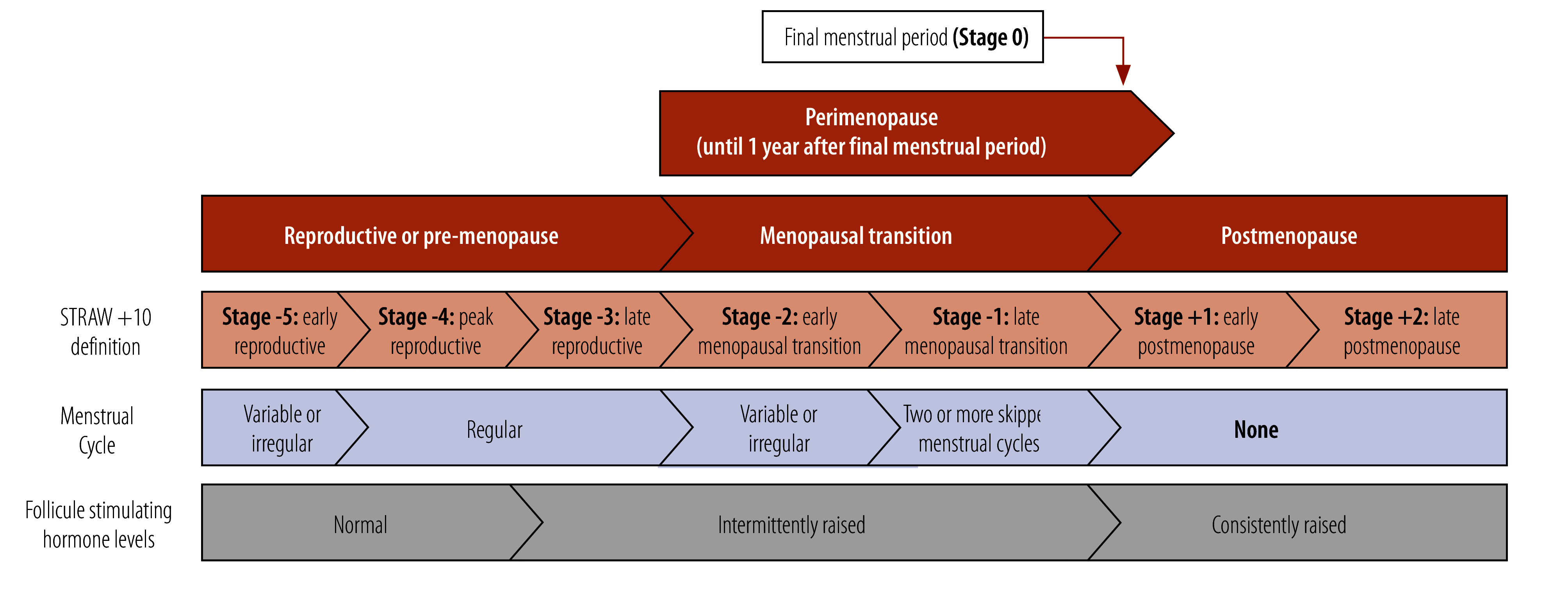
Reproductive stages with menstrual cycle and follicle stimulating hormone changes

FSH: follicle stimulating hormone; STRAW+10: Stages of Reproductive Aging Workshop.

Source: Adapted from Harlow et al., 2012.^1^

Note: the Stages of Reproductive Aging Workshop was initially completed in 2001 and then updated in 2011 as the STRAW+10 classification.

Using age as a diagnostic risk factor for perimenopause is of limited use. Research shows a wide range of both duration of perimenopause and expected age at menopause, with a higher prevalence of early or premature menopause in low- and middle-income countries compared to high-income countries.^2^ Symptoms can also have limited value in diagnosing perimenopause, with significant variations in experience across populations. Most cultures recognize hot flushes (or hot flashes) and night sweats, described as vasomotor symptoms, as a key symptom of perimenopause. However, even when only considering this singular group of symptoms, the prevalence of vasomotor symptoms is extremely variable; a systematic review of 66 papers and from all continents found rates of vasomotor symptoms varying from 3% to 86%.^3^

Considerable variation exists between parts of the world when considering other symptoms that are reported by many women in perimenopause such as cognitive issues, mood changes, sleep disturbances, urogenital symptoms, muscle and joint pain and change in libido.^4^^,^^5^ Uncertainty exists about what symptoms menopause causes; in 2005, the United States National Institutes of Health concluded that only vasomotor symptoms and vaginal dryness were clearly attributable to menopause. Similarly, another study concluded that variations between women in the experience of menopause meant there was no universal menopause syndrome.^6^

## Diverse experiences

Women’s experiences are often affected by inequities in education and workplace provisions, gender discrimination, their community’s health care, resources in their environment such as community green spaces, and more recently, by a burgeoning menopause industry that profits from those who struggle to access accurate and evidence-based information.^7^ Menopause experience, including attitudes towards perimenopause, is diverse across countries.^8^ This heterogeneity is of direct clinical importance, since prospective studies show that women entering perimenopause with more negative expectations are more likely to have troublesome symptoms.^9^ A qualitative study conducted in rural villages in Limpopo province, South Africa, found that menopause and perimenopause are often considered taboo, private or sensitive topics. Combined with the region’s high poverty and illiteracy levels, this perception leads to mental and physical health difficulties, discrimination and stigmatization, and lack of support for those experiencing perimenopause. In comparison, limited evidence from Australian First Nations women suggests that transition to menopause confers additional social status and respect.

Preparation for perimenopause occurs within and outside of formal health-care settings. Within health care, many people, due to economic, cultural, geographic or political restrictions, do not have access to any health workers, with more than half of the world’s population not covered by essential health services. Even with improved access to trained clinicians, women may not feel their symptoms are taken seriously. At the same time, clinicians may not feel adequately trained to diagnose and treat perimenopause. While many countries have clinical guidelines related to menopause, these often do not include perimenopause – and in low- and middle-income countries, fewer national guidelines are available than in high-income countries.^10^

Regarding treatment for troublesome symptoms in perimenopause, considerable variation exists in access to effective therapies. Many alternative treatment options are available globally, such as herbal remedies in Japan and traditional medicines in China, which have inconclusive or incomplete evidence for efficacy. More generally, some evidence suggests that non-pharmacological interventions such as tai-chi and cognitive behavioural therapy can help to reduce vasomotor symptoms, although conducting double-blinded randomized clinical trials with non-pharmacological interventions is challenging, and double-blinded medication trials show high placebo effects.

Outside of the health-care setting, access to information about perimenopause may be restricted due to lack of education or stigma, or because research and clinical teams are not considering specific subgroups of people such as those living with human immunodeficiency-virus or with disabilities, or gender-diverse people such as transgender men or non-binary people. Conversely, some populations are struggling to navigate an excess of information allegedly designed to help them prepare for perimenopause but including poorly evidenced solutions. Misinformation through unregulated websites and social media is increasing, and so are profit-driven commercial products ranging from misleading menopause tests to dubious supplements.

Issues that may seem specific to perimenopause often serve to highlight wider inequalities. For example, women may have difficulty meeting basic needs related to water and sanitation, affecting their ability to manage symptoms. Prolonged menstrual bleeding in perimenopause has been recorded to affect more than 90% of women on at least one occasion and nearly 80% of women on at least three occasions;^11^ where period products or treatments for heavy bleeding are unavailable or unaffordable, this bleeding may be particularly difficult to manage. In addition, heavy menstrual bleeding is the most common cause of iron deficiency anaemia worldwide, which negatively affects cognition, work ability and quality of life. 

Finally, the lack of high-quality, evidence-based information about perimenopause contributes to poor awareness of perimenopause, and is a result of less interest in women and women’s health by society and in research.^12^

## Achieving healthy menopause 

Local and international initiatives to tackle the breadth of issues facing people experiencing menopause are being implemented. Improving understanding of perimenopause will need large prospective studies focusing on multiethnic communities – such as the Study of Women’s Health Across the Nation and the Collaborative Group for Research of the Climacteric in Latin America – to be replicated in more countries and in lower-income settings.

Within health-care settings, health workers need standardized, high-quality and affordable medical education on perimenopause and menopause across all regions; we need national, regional, or even international medical curricula and guidance financed by resource-rich countries and designed by and with resource-poor countries. Regarding the education of the public, public health initiatives at national and regional levels should be funded to ensure that women everywhere have access to accurate information and support. 

Even with better awareness of their own perimenopausal symptoms, women need better access to care and solutions for their symptoms. As abnormal bleeding is so common in perimenopause, areas of the world where period poverty is common would benefit from free period products and access to clean toilets. Managing the wider symptoms and sequalae of perimenopause beyond bleeding requires women to have access to both pharmacological options and resources that enable them to undertake recommended lifestyle changes such as living in an environment that facilitates exercise. For example, a physical activity programme for menopausal women (more than half of whom had only a primary school-level education or lower) in Tabriz, Islamic Republic of Iran, significantly reduced their menopause symptoms.^13^

Recognizing the heterogeneity of the perimenopause experience, both between women and between cultures and countries, is important. Inequality is a major factor in these differences, and an individual’s experience of perimenopause is likely determined by social, cultural and religious norms, their ethnic or gender identity, their education level, and their wealth and economic power as well as their urban or rural location.

Global organizations such as the World Health Organization have a key role in leading efforts to reduce health inequalities in perimenopause. Improvements can be made at every stage of a woman’s journey, from her education to access to empathetic and knowledgeable health workers, and her access to safe, effective management options for troublesome symptoms.
